# Cocoa extract intake for 4 weeks reduces postprandial systolic blood pressure response of obese subjects, even after following an energy-restricted diet

**DOI:** 10.3402/fnr.v60.30449

**Published:** 2016-03-31

**Authors:** Idoia Ibero-Baraibar, Manuel Suárez, Anna Arola-Arnal, M. Angeles Zulet, J. Alfredo Martinez

**Affiliations:** 1 Department of Nutrition, Food Science and Physiology, University of Navarra, Pamplona, Spain; 2 Centre for Nutrition Research, Faculty of Pharmacy, University of Navarra, Pamplona, Spain; 3 Nutrigenomics Research Group, Department of Biochemistry and Biotechnology, Rovira i Virgili University, Tarragona, Spain; 4 Centre Tecnològic de Nutrició i Salut (CTNS), TECNIO, CEICS, Reus, Spain; 5 Physiopathology of Obesity and Nutrition, CIBERobn, Carlos III Health Research Institute, Madrid, Spain; 6 Navarra Institute for Health Research (IdiSNA), Pamplona, Spain

**Keywords:** blood pressure, cocoa, diet, bioavailability, polyphenols, weight loss

## Abstract

**Background:**

Cardiometabolic profile is usually altered in obesity. Interestingly, the consumption of flavanol-rich foods might be protective against those metabolic alterations.

**Objective:**

To evaluate the postprandial cardiometabolic effects after the acute consumption of cocoa extract before and after 4 weeks of its daily intake. Furthermore, the bioavailability of cocoa extract was investigated.

**Design:**

Twenty-four overweight/obese middle-aged subjects participated in a 4-week intervention study. Half of the volunteers consumed a test meal enriched with 1.4 g of cocoa extract (415 mg flavanols), while the rest of the volunteers consumed the same meal without the cocoa extract (control group). Glucose and lipid profile, as well as blood pressure and cocoa metabolites in plasma, were assessed before and at 60, 120, and 180 min post-consumption, at the beginning of the study (Postprandial 1) and after following a 4-week 15% energy-restricted diet including meals containing or not containing the cocoa extract (Postprandial 2).

**Results:**

In the Postprandial 1 test, the area under the curve (AUC) of systolic blood pressure (SBP) was significantly higher in the cocoa group compared with the control group (*p*=0.007), showing significant differences after 120 min of intake. However, no differences between groups were observed at Postprandial 2. Interestingly, the reduction of postprandial AUC of SBP (AUC_Postprandial 2-AUC_Postprandial 1) was higher in the cocoa group (*p*=0.016). Furthermore, cocoa-derived metabolites were detected in plasma of the cocoa group, while the absence or significantly lower amounts of metabolites were found in the control group.

**Conclusions:**

The daily consumption of cocoa extract within an energy-restricted diet for 4 weeks resulted in a greater reduction of postprandial AUC of SBP compared with the effect of energy-restricted diet alone and independently of body weight loss. These results suggest the role of cocoa flavanols on postprandial blood pressure homeostasis.

Obesity is considered one of the major public health problems associated with cardiovascular mortality (1). In that situation, different fasting and postprandial metabolic markers are altered contributing to the development of the obesity-associated comorbidities such as diabetes, insulin resistance, hypertension, atherosclerosis, and dyslipidaemia, among others (2, 3). Interestingly, the prescription of nutritional strategies as well as lifestyle changes, such as the reduction of energy intake and adherence to healthy dietary patterns, reduces the risk of suffering from cardiometabolic disorders (4, 5). On the other hand, the intake of plant extracts, which are rich on polyphenols, is receiving especial attention in the protection against obesity-associated comorbidities (6). In this context, cocoa is one of the richest sources of polyphenols with claimed benefits on blood pressure (7, 8), insulin resistance (7, 9), lipid profile (9–11), endothelial dysfunction or oxidative stress (10, 12–14), and inflammation (9, 15–18). Such therapeutic effects have been attributed to some of the bioactive compounds occurring in cocoa, mainly flavanols, which are the most abundant polyphenols in this seed (19). Flavanols in cocoa are found as monomers ((−)-epicatechin and (+)-catechin) and procyanidins (20). Furthermore, cocoa also contains other bioactive compounds such as methylxanthines (caffeine and theobromine) and minerals (magnesium, copper, iron, etc.) with potential healthy properties (21).

In order to establish a relationship between cocoa consumption and healthy benefits through physiological mechanisms, flavanols from cocoa need to be absorbed into the circulation (22). Bioavailability depends on different factors such as food matrix, physical state, and the degree of flavanol polymerisation (23). Cocoa monomeric and some oligomeric flavanols are stable in the stomach and small intestine (24, 25) and are rapidly absorbed appearing in plasma between 30 and 60 min post-consumption (20, 26). Afterwards, glucoronidation, sulphation, and methylation of the monomeric flavanols in the liver and in the small intestine produce *O*-glucoronidated, *O-*sulphated, and *O*-methylated flavanol derivates in plasma (23). Once in the bloodstream, these metabolites undergo additional conjugations in the liver and returned back to the small intestine by enterohepatic circulation (23). Procyanidins are poorly absorbed; only procyanidin dimer B2 has been detected in human plasma (24, 25). Unabsorbed flavanols reach the colon where after the degradation by the intestinal microbiota and the transformations to phenolic acids are then absorbed into the circulation (27). In addition, phase II metabolites are excreted into the bile where after the bacterial enzyme activities are reabsorbed into the circulation. Finally, metabolites are transferred from the bloodstream to the kidneys to be excreted in urine (23).

On the other hand, the intake of a food component could have a different effect when consumed first time or when consumed regularly during a determined period of time (28). Surprisingly, it has not yet been well documented if the cardiometabolic response to the acute consumption of cocoa extract could be persistent with time or it could be influenced by its regular consumption during a determined period of time, resulting in an adaptive or tachyphylactic effect.

The present research is a substudy carried out within a clinical trial (NCT01596309), whose principal purpose was to evaluate the effect of consuming ready-to-eat meals containing cocoa extract under a moderate energy–restricted diet for 4 weeks on the general nutritional status, as well as on cardiometabolic and oxidative markers of middle-aged obese subjects. The obtained results evidenced the improvement of oxidised low-density lipoprotein cholesterol levels (13). In addition, the prescribed energy-restricted diet reduced the adiposity as well as improved blood pressure, routine blood biochemical profile, and 25-hydroxyvitamin D levels (29, 30).

The aim of the present study was specifically focussed on analysing the postprandial response of the acute consumption of cocoa extract during the immediate 3 h of intake on routine blood biochemical and blood pressure markers before and after 4 weeks of its daily consumption. Furthermore, the bioavailability of the cocoa extract within the ready-to-eat meals provided in the study was evaluated measuring cocoa-derived metabolites in plasma.

## Methods

### Subjects

**Fig. 1:**
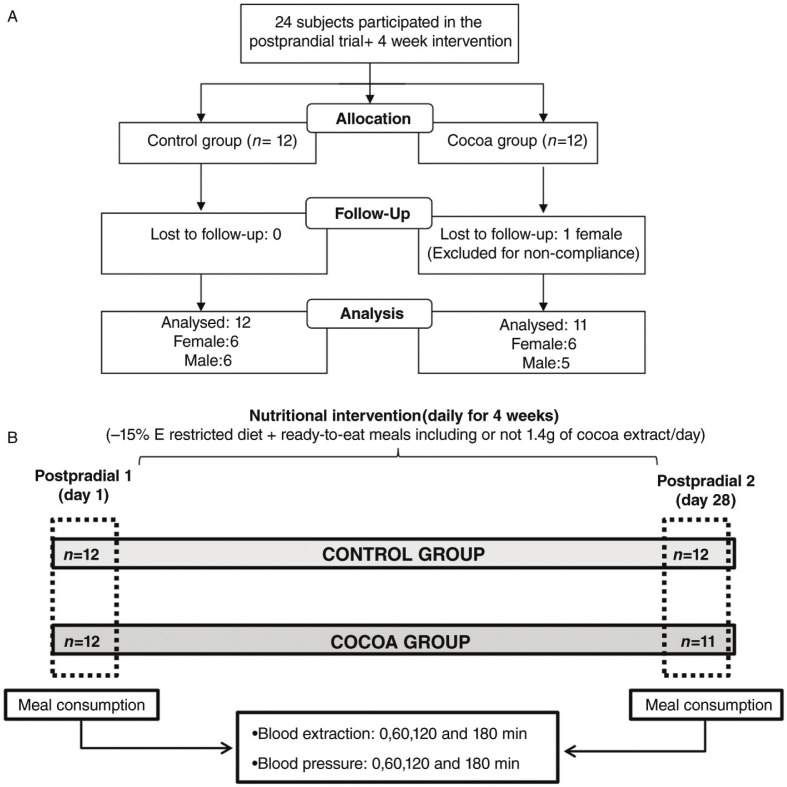
A) Flow chart of the participants. B) Study diagram.

From the main study (*n*=50), 24 volunteers (58.2 (5.2) years) took part in the present substudy: 12 allocated in the control group (6 men and 6 women) and the remaining 12 in the cocoa group (5 men and 7 women) as shown in Fig. 1A.

All the participants gave written informed consent to participate in the trial. To be enrolled in the study subjects needed to be between 50 and 80 years old, with a body mass index (BMI) of 27.0–35.5 kg/m^2^ and maintaining a stable weight (<5% of variation) within the previous 3 months to the intervention. Subjects suffering from the following conditions were not included: gastrointestinal disease, hepatic diseases, diabetes, cancer or inflammatory disease, food allergies, or cognitive and psychiatric alterations. Also, individuals following a weight loss treatment, hormone replacement, anti-inflammatory, hypocholesterolaemic or blood pressure lowering treatments were not allowed to participate. Subjects taking antidepressant drugs, antioxidant-rich supplements, medication that could influence appetite or nutrient absorption, inability to follow-up the intervention and smokers were also excused from the study.

### Study design

The study was carried out in the Metabolic Unit of the University of Navarra (Pamplona, Spain). The trial was approved by the Research Ethics Committee of the University of Navarra (ref. no 006/2012) and followed the Helsinki Declaration guidelines. Furthermore, it was registered at www.clinicaltrials.gov (NCT01596309). CONSORT guidelines 2010 were considered.

One week before the beginning of the intervention, volunteers were required to exclude cocoa and cocoa-containing products from their habitual diet. In addition, 3 days before the beginning of the study, all of the volunteers were required to follow a low-polyphenolic diet which was restricted in fruits and vegetables and excluded polyphenol rich foods such as cocoa, coffee, tea, infusions, botanical or antioxidant-rich supplements as well as juices and alcoholic drinks.

The postprandial study was performed on the first day (Postprandial 1) and the last day (Postprandial 2) of a 4-week nutritional intervention (Fig. 1B). The procedure consisted of the consumption of a test meal (a ready-to-eat dish and a dessert) containing 1.4 g of cocoa extract, or the same meal without cocoa extract, depending on the group allocation. The experiment was carried out after a 10-h overnight fast. Blood samples and blood pressure measurements were taken just before the consumption of the test meal (time 0) and at 60, 120 and 180 min post-consumption. The postprandial test was repeated after 4 weeks (day 28) of following an energy-restricted diet (−15%E) including or not including the daily consumption of ready-to-eat meals containing 1.4 g/day of cocoa extract, depending on the experimental group allocation (Fig. 1B). The energy-restricted diet consisted of 45% of the total caloric value from carbohydrates, <30% from lipids and 22–25% from proteins, as explained elsewhere (13).

The randomisation to participate in the main study was performed using the ‘random between 1 and 2’ function in the Microsoft Office Excel (Microsoft Iberica, Madrid, Spain). Boxes in which the meals were provided had the same appearance and differed only on the code label, ensuring the double-blind protocol. The dietary restriction of foods containing cocoa and polyphenol rich foods was maintained during the 4 weeks of intervention and volunteers were also asked not to change their physical activity patterns. Three days before the end of the intervention, volunteers were prescribed to consume predetermined types of test meals from the variety of meals they had available for the last week of the intervention, in order to make all the volunteers reach the endpoint of the study in similar nutritional conditions avoiding nutritional interferences.

### Test meals

The postprandial test included a meal containing 1.4 g of cocoa extract or the same meal without cocoa extract, which was based on a ready-to-eat dish and dessert: courgette cream (300 g) and chocolate custard (150 g). The whole meal provided a total energy of 365 kcal and was composed of (in grams and proportions (%)) 34.2 g (7.6%) of carbohydrates, 20.9 g (4.6%) of lipids, 8.9 g (2.0%) of proteins, 4.9 g (1.1%) of fibre, 377.7 g (83.9%) of water, and 4.1 g (0.9%) of ash.

### Characterisation of cocoa extract

The cocoa extract and the analytical characterisation were supplied by Nutrafur S.A. (Murcia, Spain). The composition of 1.4 g of cocoa extract as mean (SD) was as follows: 140.4 (7.1) mg of theobromine, 645.3 (32.3) mg of total polyphenols as catechin, 414.3 (20.7) mg of flavanols as catechin, 153.4 (7.7) mg of epicatechin, 14.6 (0.7) mg of catechin, 99.4 (5.0) mg of procyanidin B2, 13.4 (0.7) mg of procyanidin B1, and 133.5 (6.7) mg of oligomeric procyanidins. Total polyphenol content was determined by Folin–Ciocalteu method, and flavanoids and theobromine were analysed by high-performance liquid chromatography (HPLC), whose analytical procedures have been described in detail elsewhere (13).

### Body weight and blood biochemical analyses

Body weight was measured underwear in an overnight fasting condition using dual-energy X-ray absorptiometry following manufacturer′s instructions (Lunar Prodigy, software version 6.0, Madison, WI). Blood samples were drawn before (time 0) and after the consumption of tested meals (60, 120 and 180 min) through an intravenous catheter inserted into an antecubital vein using ethylenediaminetetraacetic acid (EDTA) and CLOT tubes (BD Vacutainer^®^). After each extraction, samples were centrifuged in order to obtain plasma and serum aliquots (15 min, 1,500 g, 4°C), which were then stored at −80 °C until analysis. Plasma glucose, total cholesterol (Total-c), and high-density lipoprotein cholesterol (HDL-c) were measured by colorimetric procedures in a Pentra C200 autoanalyser (Horiba Medical, Montpellier, France).

### Blood pressure determination

Blood pressure was determined immediately before the consumption of the test meal (time 0) and at 60, 120 and 180 min post-consumption using an automatic monitor device (Intelli Sense. M6, OMRON Healthcare, Hoofddorp, the Netherlands). Measures were carried out always in the same arm, sitting on a chair with the cuff at the same level as the heart and with the arm in relaxed position. Between measurements, volunteers were maintained in resting conditions, free of any alterations in a quiet and temperature-controlled room.

### Analysis of cocoa extract derived metabolites

The assessment of cocoa-derived metabolites in plasma was performed to evaluate the bioavailability of cocoa extract and to assess the adherence of the volunteers to the intervention, as well as to compare the metabolite concentration in plasma before and after 4 weeks of daily cocoa consumption. Fourteen cocoa-derived metabolites were targeted in plasma of cocoa group in the Postprandial 1 test and the same metabolites were measured in the Postprandial 2 in cocoa and control groups. The analysed metabolites were: catechin, epicatechin, methyl-epicatechin-glucuronidate, procyanidin B2, methyl-catechin-glucuronidate, catechin-sulphate, epicatechin-glucuronidate, epicatechin-sulphate, 3-*O*-methyl-epicatechin, methyl-epicatechin-*O*-sulphate, 3,7-dimethyluric acid, 1-methylxanthine, 3-methylxanthine, and theophylline.

#### Chemicals and reagents

Procyanidin B2 was purchased from Extrasynthese (Genay, France). (+)-catechin and (−)-epicatechin were purchased from Fluka Co. (Buchs, Switzerland) while 3-methylxanthine and 3,7-dimethyluric acid were from Sigma Aldrich (St. Louis, MO). Cathecol was used as internal standard (IS). Methanol (HPLC grade), acetonitrile (HPLC grade) and acetic acid were all provided by Scharlau Chemie (Barcelona, Spain). Ortho-phosphoric acid (85%) was purchased from Panreac (Barcelona, Spain). Water was of milli-Q quality (Millipore Corp, Bedford, MA).

#### Phenolic metabolites’ extraction

Phenolic metabolites were extracted by using microelution plates (Waters, Milford, MA) packed with 2 mg of OASIS HLB sorbent (Waters) following the method described by Serra et al. (31) with minor modifications. Briefly, the wells were sequentially conditioned by using 250 µL of methanol and 250 µL of milli-Q water:acetic acid (99.8:0.2, v/v). Then, 200 µL of plasma mixed with 350 µL of phosphoric acid 4% and 50 µL of catechol (IS) at 0.5 mg/L were loaded into the wells. After that, the clean-up of the plates was sequentially done with 200 µL of milli-Q water and 200 µL of milli-Q water:acetic acid (99.2:0.2, v/v) to eliminate any interference that the sample might contain. Finally, the elution of the retained metabolites was performed with 2×50 µL of acetone:milli-Q water:acetic acid (70:29.5:0.5, v/v/v). A 5 µL portion of the eluted metabolites was directly injected into the HPLC–MS/MS (Agilent Technologies, Palo Alto, U.S.A.).

#### Theophylline metabolites extraction

Theophylline metabolites were extracted following the method described by Ogawa et al. (32) with some minor modifications. Thus, 50 µL of plasma was mixed with 100 µL of acetonitrile, vortexed for 1 min and centrifuged for 5 min at 1,000 rpm. Pellets were removed, and solvent was evaporated to dryness under nitrogen flow rate. Finally, samples were reconstituted with 50 µL of milli-Q water:acetic acid (99.9:0.1, v/v) and were analysed by HPLC-MS/MS.

#### HPLC–MS/MS measurements

The HPLC–MS/MS system consisted of an Agilent 1200 Series instrument (Agilent Technologies, Palo Alto, CA) using a Zorbax SB-Aq column (3.5 µm, 150 mm×2.1 mm i.d.) equipped with a Pre-Column Zorbax SB-C18 (3.5 µm, 15 mm×2.1 mm i.d.) and coupled to a triple quadrupole 6410 also from Agilent.

Two chromatographic methods were used to analyse the whole range of metabolites. To carry out the analysis of phenolic metabolites the column was kept at 25°C and the flow rate was 0.4 mL/min. The composition for solvent A was milli-Q water:acetic acid (99.8:0.2 v/v) while for solvent B, acetonitrile. The elution gradient was 0–10 min, 5–55% B; 10–11 min, 55–80% B; 11–12 min, 80% B; 12–13 min, 80–5% B; and 5 min post-time. On the other hand, to analyse theophylline metabolites, milli-Q water:acetic acid (99.8:0.2 v/v) was used as solvent A and acetonitrile as solvent B. The elution gradient was 0–2 min, 3–10% B; 3–4 min, 10–50% B; 4–5 min, 50–95% B; 5–6 min, 95% B; 6–6.5 min, 95–3% and 2 min post-time. The flow rate was held at 0.5 mL/min throughout all the run time.

Ionisation was performed by electrospray (ESI) in the negative mode and the source parameters were drying gas temperature 350°C, flow rate 12 L/min, gas nebuliser pressure 45 psi, and the capillary voltage 4,000 V. The selected reaction monitoring (SRM) transitions and the instrumental parameters for each compound are reported (Supplementary Table 1). Due to the lack of standards of some metabolites, they were tentatively quantified by using the calibration curves corresponding to their phenolic precursors as is specified in Supplementary Table 2. Quality parameters are reported (Supplementary Table 2). Results are expressed as nmol/L of each metabolite/compound in the plasma sample. Values under the limit of quantification (LOQ) are expressed as ‘n.q.’ (not quantified), while values under the limit of detection (LOD) are showed as ‘n.d.’ (not detected).

### Statistical analyses

Data were analysed using the SPSS 15.0 for Windows statistical program (SPSS, Inc., Chicago, IL) considering results with *p* values less than 0.05 as statistically significant. Normality of the variables was assessed by the Shapiro–Wilk test. Data are expressed as mean (SD). Comparisons between baseline and the endpoint of the intervention were analysed by the student paired *t* test or the Wilcoxon test. The comparisons between both groups were performed by parametric *t* test or Mann–Whitney U test, depending on the normality of the variables. Area under the curve (AUC) was calculated for blood biochemical markers, blood pressure, and cocoa extract–derived metabolites in plasma according to the trapezoid rule (33) in GraphPad Prism version 5 for Windows (GraphPad Software, San Diego, CA). Thus, the AUC was determined per hour, and then the sum of the 3 AUCs was performed. For cocoa metabolites, the AUC was calculated when at least the metabolites were quantifiable at one time-point. However, to perform the statistical analysis and to calculate the *p* value derived from the comparison of AUC of control and cocoa groups, the original data of each participant was used, even if it was under the LOQ or LOD. Analysis of covariance (ANCOVA) was performed to compare groups in the Postprandial 1and Posprandial 2 adjusted for the baseline value of each variable in each test and baseline weight or weight change (Δ=endpoint-baseline) when appropriate. The comparisons of the AUC changes (Postprandial 2 − Postprandial 1) between groups were adjusted for the changes of each assessed variable at fasting and Δweight. Covariates were included in the ANCOVA analysis to avoid results bias. The comparisons between Postprandial 1 and Postprandial 2 at different time points were evaluated by repeated measures analysis of variance (ANOVA) for multiple comparisons by Bonferroni correction. Although some metabolites were under the LOQ or LOD, repeated measures ANOVA were performed with those values when appropriate.

## Results

### Subjects

Twenty-three subjects of the initial twenty-four [58.2 (5.2) years] completed the study. A woman from the cocoa group was removed due to dietary non-compliance (self-reported), and hence 11 subjects finished the study under cocoa treatment (Fig. 1A). This volunteer was also excluded from the statistical analyses following per protocol criteria. Although differences were observed at baseline in several of the assessed variables, none of these were at statistically significant levels (Table 1). As expected, body weight was significantly reduced in both intervention groups, control: −2.8 (1.3) and cocoa: −2.7 (0.9), after the 4-week intervention period, without statistical differences between groups.

**Table 1:** Anthropometric and clinical characteristics of subjects from control and cocoa groups at baseline

	Baseline		
		
Variables	Control (*n*=12)	Cocoa (*n*=11)	p^a^
Age	57 (4.9)	59 (5.4)	0.332
Weight (kg)	83.5 (9.8)	83.3 (10.9)	0.970
BMI (kg/m^2^)	30.2 (2.2)	31.4 (2.6)	0.258
Waist (cm)	103.7 (4.8)	105.2 (6.8)	0.523
SBP (mmHg)	116 (14.4)	122 (16.5)	0.303
DBP (mmHg)	76 (8.9)	80 (7.1)	0.326
Glucose (mg/dL)	96.2 (8.6)	99.1 (7.0)	0.387
Insulin (µU/mL)	7.6 (5.1)	9.2 (7.4)	0.559
Total-c (mg/dL)	220.6 (37.4)	237.0 (64.8)	0.460
HDL-c (mg/dL)	55.5 (15.2)	52.2 (8.2)	0.533

Data presented as mean (SD).

a Comparison between control and cocoa groups.

*p*<0.05 was considered significant.

BMI: body mass index; DBP: diastolic blood pressure; HDL-c: high-density lipoprotein cholesterol; SBP: systolic blood pressure; Total-c: total cholesterol.

### Postprandial glucose and lipid metabolism response

Differences between groups were not found, neither in the AUC of glucose nor in the lipid metabolism variables during Postprandial 1 and Postprandial 2 tests. Both groups reported lower AUC of total cholesterol and HDL-c at Postprandial 2 (Table 2), but no differences were observed when the AUC changes (Postprandial 2 − Postprandial 1) were compared between groups (Table 2).

**Table 2:** Area under the curve of biochemical and blood pressure variables at Postprandial 1 and 2

	Postprandial 1 (day 1)			Postprandial 2 (day 28)					
					
AUC	Control (*n*=12)	Cocoa (*n*=11)	*p*^a^	Control (*n*=12)	Cocoa (*n*=11)	*p*^b^	*p*^c^	*p*^d^	*p*^e^
Glucose (mg·h/dL)	286.9 (43.5)	314.8 (29.1)	ns	292.9 (37.3)	314.4 (33.6)	ns	ns	ns	ns
Insulin (µU·h/dL)^f^	75.9 (60.4)	97.6 (73.8)	ns	68.6 (50.3)	77.0 (48.5)	ns	ns	ns	ns
Total-c (mg·h/dL)	664.5 (120.2)	714.1 (194.7)	ns	551.0 (88.1)	577.2 (138.8)	ns	<0.001	0.001	ns
HDL-c (mg·h/dL)	161.7 (44.0)	154.0 (25.4)	ns	141.0 (25.6)	134.3 (23.8)	ns	0.011	0.001	ns
SBP (mmHg·h)	338.0 (31.0)	376.0 (46.6)	0.007	317.0 (28.8)	340.0 (36.6)	ns	0.015	0.001	0.016
DBP (mmHg·h)	226.0 (19.8)	240.0 (22.8)	ns	205.0 (17.9)	220.0 (21.7)	ns	<0.001	0.001	ns

Data presented as unadjusted mean (SD). Comparisons between both groups were performed with an independent *t* test or Mann–Whitney U test and comparisons between Postprandial 1 and 2 were analysed by paired student *t* test or Wilcoxon test, depending on the normality of the variables.

ANCOVA univariate analyses adjusted for fasting value of each assessed variable, baseline weight, and ▵weight differently combined were performed.

*p*<0.05 was considered as significant.

a AUC control vs. AUC cocoa group in Postprandial 1, adjusted for baseline value of the assessed variable in Postprandial 1 and baseline weight.

b AUC control vs. AUC cocoa group at Postprandial 2, adjusted for baseline value of the assessed variable at Postprandial 2 and Δweight.

c AUC control group in the Postprandial 1 vs. AUC control group in the Postprandial 2.

d AUC cocoa group in the Postprandial 1 vs. AUC cocoa group in the Postprandial 2.

e Difference in the change (Postprandial 1 and Postprandial 2) in AUC between control and cocoa groups, adjusted for Δbaseline value of the variable (baseline value at Postprandial 2-fasting value at Postprandial 1) and Δweight.

f No-normally distributed variables.

AUC: area under the curve; DBP: diastolic blood pressure; HDL-c: high-density lipoprotein cholesterol; ns: not significant; SBP: systolic blood pressure; Total-c: total cholesterol; Δweight: weight change.

### Postprandial blood pressure response

**Fig. 2:**
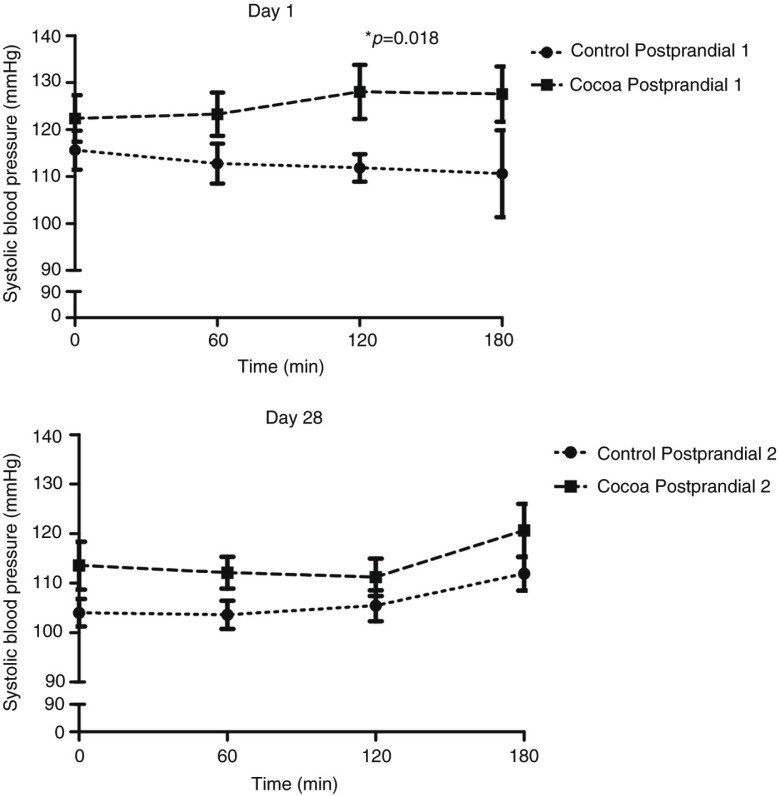
Systolic blood pressure before (time 0) and after meal consumption (60, 120, and 180 min) in control (*n*=12) and cocoa (*n*=11) groups.

**Fig. 3:**
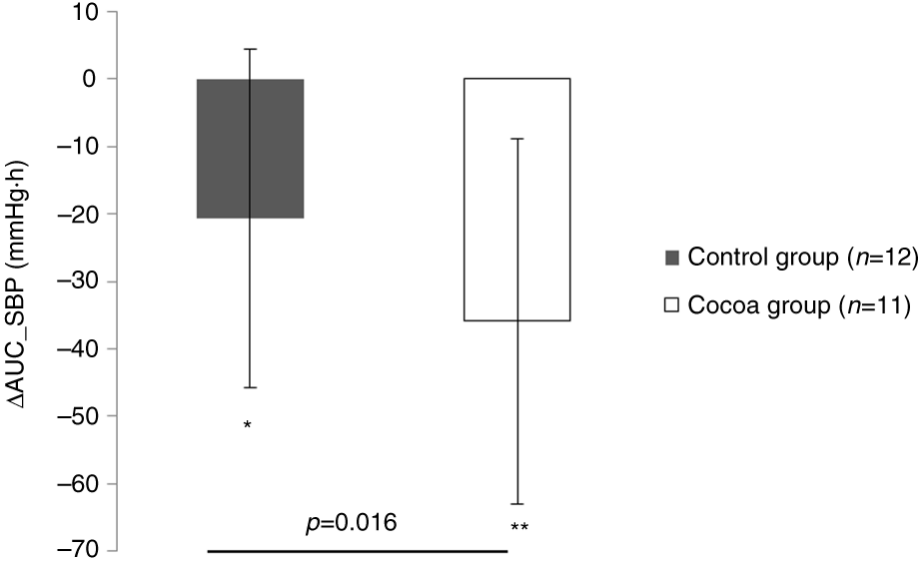
Comparison between groups of the change of area under the curve (AUC) of systolic blood pressure (Postprandial 2 − Postprandial 1).

The AUC concerning SBP was significantly (*p*=0.007) higher in the cocoa group compared with the control group during the Postprandial 1 adjusted for baseline SBP and baseline weight (Table 2). Specifically, repeated measures analysis revealed that the consumption of the cocoa-supplemented meal resulted in a significantly higher (*p*=0.018) SBP levels at 120 min post-consumption when compared to control group (Fig. 2). However, no differences were found along time in the SBP within the cocoa or control groups in the Postprandial 1. In the Postprandial 2, no statistical differences concerning AUC of SBP were found between groups adjusted for baseline SBP in the Postprandial 2 and weight change (Table 2). When the AUC of Postprandial 1 and Postprandial 2 was compared within each group, the AUC of SBP decreased significantly in both groups, (control *p*=0.015 and cocoa *p*=0.001), but interestingly, the reduction of AUC (Postprandial 2 − Postprandial 1) in the cocoa group was significantly higher (*p*=0.016) in comparison to the control group when controlled for the change of baseline SBP and weight change during the 4 weeks (Fig. 3).

Concerning the AUC of DBP, no differences were noted between groups neither in the Postprandial 1 nor in the Postprandial 2 (Table 2). When the AUC of DBP was compared within the same group between Postprandial 1 and 2, significant reduction was observed in both groups (control *p*<0.001, cocoa *p*=0.001). However, when the AUC changes (Postprandial 2 − Postprandial 1) for DBP were compared between groups, no significant differences were found (Table 2).

### Cocoa-derived metabolites in plasma

Fourteen metabolites related with cocoa intake were measured in plasma of cocoa group at Postprandial 1 and in plasma of control and cocoa groups at Postprandial 2. Data are presented when at least in one of the groups the metabolite was quantifiable (Table 3). Metabolites were monomeric and dimeric flavanols (catechin, epicatechin, and procyanidin B2), alkaloid metabolites (3-methylxanthine, 1-methylxanthine, 3,7-dimethyluric acid, and theophylline) and flavanol phase II metabolites (methyl-epicatechin-glucuronidate, methyl-catechin- glucuronidate, catechin-sulphate, epicatechin- glucuronidate, epicatechin-sulphate, methyl-epicatechin-*O*-sulphate, and 3-*O*-methyl-epicatechin).

**Table 3:** Cocoa-derived metabolites in plasma before (time 0) and after meal consumption (60, 120, and 180 min)

	Time (min)				*p*^a^				
				
Cocoa metabolites (nmol/L)	0	60	120	180	*p*_0–180′_	*p*_P1–P2_	*p* _0–180′_ _×__P1–P2_	AUC (nmol·h/L)	*p* AUC^b^
**Procyanidin B2**^e^									
Cocoa-P1	n.d.	3.2 (1.8)	3.1 (1.1)	n.q.	<0.001	n.s.	n.s.	7.6 (3.1)	ns
Cocoa-P2	n.d.	3.1 (1.3)	n.q.	n.q.				7.3 (3.7)	
Control-P2	n.d.	n.d.	n.d.	n.d.				–	
**Methyl-catechin-glucuronidate**									
Cocoa-P1	n.d.	42.6 (23.9)	43.2 (12.8)	39.2 (11.9)	<0.001	n.s.	n.s.	105.9 (38.2)	ns
Cocoa-P2	n.q.	55.2 (18.0)	62.6 (45.1)	41.4 (12.8)				143.7 (58.87)	
Control-P2	n.d.	n.q.	n.q.	n.q.				–	
**Epicatechin-glucuronidate**									
Cocoa-P1	n.d.	1,346.8 (677.8)	1,267.6 (499.7)	968.1 (360.7)	<0.001	n.s.	n.s.	3,098.4 (1,234.3)	ns
Cocoa-P2	104.4 (63.9)	1,576.5 (478.9)	1,371.1 (366.4)	998.6 (388.1)				3,499.2 (955.0)	
Control-P2	14.4 (29.9)	40.6 (22.3)	44.4 (21.9)	63.9 (101.3)				124.2 (82.4)	
**Catechin-sulphate**^e^									
Cocoa-P1	n.d.	133.7 (53.3)	106.0 (40.1)	65.8 (24.2)	<0.001	n.s.	n.s.	272.6 (85.6)	ns
Cocoa-P2	12.1 (17.0)	138.1 (51.0)	106.8 (48.7)	62.6 (45.9)				282.2 (110.2)	
Control-P2	n.d.	n.d.	n.d.	n.d.				–	
**Methyl-epicatechin-*O*-sulphate**									
Cocoa-P1	n.d.	353.7 (138.5)	353.3 (111.7)	276.9 (59.7)	<0.001	n.s.	n.s.	845.4 (249.0)	ns
Cocoa-P2	24.2 (12.1)	397.5 (108.4)	385.6 (79.8)	284.6 (80.9)				937.5 (185.9)	
Control-P2	n.q.	n.d.	13.9 (4.0)	16.5 (26.1)				36.3 (19.8)	
**Epicatechin-sulphate**									
Cocoa-P1	n.d.	2,847.6 (1,034.2)	2,970.6 (958.2)	2,074.4 (524.2)	<0.001	n.s.	n.s.	6,734.1 (2,164.3)	ns
Cocoa-P2	152.2 (98.8)	3,311.2 (1,029.2)	3,374.3 (996.0)	2,201.2 (885.0)				7,862.1 (1,862.2)	
Control-P2	n.q.	92.2 (42.6)	80.0 (20.8)	107.0 (175.7)				229.7 (135.1)	
**3-*O*-methyl-epicatechin**									
Cocoa-P1	n.d.	137.8 (51.8)	136.6 (47.0)	92.2 (37.9)	<0.001	n.s.	n.s.	320.5 (104.2)	ns
Cocoa-P2	n.q.	151.1 (40.7)	147.8 (33.3)	110.1 (36.6)				358.2 (76.6)	
Control-P2	n.d.	n.q.	n.q.	n.q.				–	
**3,7-dimethyluric acid**									
Cocoa-P1	n.d.	66.1 (138.3)	n.q.	n.q.	0.002	n.s.	n.s.	135.4 (171.8)	ns
Cocoa-P2	n.q.	68.0 (43.4)	61.9 (32.1)	77.6 (82.7)				176.8 (117.4)	
Control-P2	n.d.	n.q.	n.q.	n.q.				–	
**3-methylxanthine**									
Cocoa-P1	n.d.^c^	458.3 (190.8)^c^	634.0 (146.8)^c^	705.5 (158.3)^c^	<0.001	<0.001	n.s.	1,445.0 (328.1)	<0.001
Cocoa-P2	881.1 (310.9)^d^	1,301.1 (394.5)^d^	1,386.4 (427.7)^d^	1,560.9 (528.1)^d^				3,908.5 (1,146.7)	
Control-P2	335.2 (303.5)	542.2 (315.7)	592.7 (331.4)	680.0 (412.8)				1,642.5 (983.8)	
**Theophylline** e									
Cocoa-P1	121.3 (212.3)^c^	285.5 (190.2)^c^	360.7 (143.3)^c^	391.0 (164.9)^c^	<0.001	0.013	n.s.	902.3 (512.3)	0.001
Cocoa-P2	726.2 (742.8)^d^	858.7 (563.9)^d^	854.4 (689.4)^d^	867.4 (555.7)^d^				2,509.8 (1,876.9)	
Control-P2	339.7 (341.3)	398.7 (328.7)	407.9 (291.1)	415.5 (329.8)				1,184.2 (949.0)	

Data presented as unadjusted mean (SD). When a mean value was not quantifiable or no detectable in control group at 0, 60, 120, or 180 min, the real values were used, although been under the limit of quantification or detection, to perform statistical analysis where appropriate. AUC was calculated when at least the metabolite was above the limit of quantification in one time-point. In those cases, also the corresponding *p*_AUC_ is reported. *p*_AUC_ was calculated using the original values of each participant where appropriate. Comparisons between P1 and P2 were analysed by paired student *t* test or Wilcoxon test, according to the normality of the variables. *p*<0.05 was considered as significant.

– Not possible to calculate AUC.

a *p* values of the repeated measures ANOVA.

b AUC of cocoa group at Postprandial 1 vs. AUC of cocoa group at Postprandial 2.

c,d Cocoa group at Postprandial 1 vs. Cocoa groups at Postprandial 2 at each time-point by multiple comparison by Bonferroni correction.

e Non-normally distributed variables.

AUC: area under the curve; n.d: not detected; n.s.: not significant; n.q.: not quantified; P1: Postprandial 1; P2: Postprandial 2.

Except theophylline, cocoa-derived metabolites were not detected in fasting condition (time 0) at the beginning of the study. Interestingly, cocoa-derived metabolites were detected in plasma samples of cocoa consumers after the meal consumption in both postprandial trials, while in the control group the absence or significantly lower concentration of those metabolites was found in the Postprandial 2 (Table 3).

As expected, a significant variation was noted along the 180 min post-consumption in the cocoa-derived metabolites. The highest concentration of metabolites was found at 60 and 90 min post-consumption (Table 3). The repeated measures ANOVA revealed no differences in the amount of metabolites between Postprandial 1 and Postprandial 2 except for 3-methylxanthine and theophylline, which presented higher amounts in the Postprandial 2 (Table 3). Similarly, when the AUC of Postprandial 1 and Postprandial 2 was compared between groups, significantly higher AUC of 3-methylxanthine (*p*<0.001) and theophylline (*p*=0.001) was detected in the second postprandial test (Table 3).

## Discussion

In this study, the postprandial effect of consuming ready-to-eat meals containing a cocoa extract was studied before and after 4 weeks of following a moderate hypocaloric diet including the daily consumption of ready-to-eat meals supplemented with cocoa extract. One of the major findings of this trial was the different postprandial response of SBP to the acute intake of cocoa extract before and after the daily consumption of cocoa extract for 4 weeks. In the Postprandial 1, the intake of cocoa extract resulted in a significantly higher acute AUC of SBP, showing significant differences at 120 min post-consumption when compared to the control group. Although none of the differences at baseline were at statistically significant levels, it cannot be discarded that the differences of baseline SBP between both groups could influence the result in some manner. However, to minimise that influence, analyses were adjusted for baseline SBP levels when appropriate as reported in Table 2. On the other hand, when the postprandial test was repeated after the daily consumption of cocoa extract for 4 weeks, the effect was not maintained, suggesting an adaptive process of the postprandial SBP when cocoa is acutely consumed after a regular intake during a short period of time. Nevertheless, the present results should be viewed with caution because although significant differences were observed respect to the control group, no significant differences between baseline SBP and the SBP at 120 min of consuming the cocoa extract were found. Most of the reports showing a significant effect on SBP after a nutritional intervention have been observed in subjects suffering from hypertension (34, 35). However, cocoa is associated with blood pressure–lowering properties, and indeed there are studies reporting a reduction of SBP levels after an intervention with cocoa in normotensive individuals. In this sense, Heiss et al. compared the effect of cocoa intake between young (*n*=22) and elderly (*n*=20) normotensive subjects reporting a significant reduction of blood pressure in elderly group (36). Similarly, Grassi et al. observed that in a 1-week crossover study the supplementation with different doses of cocoa resulted in a significant reduction of SBP in overweight normotensive subjects (37). On the other hand, studies involving the acute administration of cocoa have reported a reduction (38, 39) or no changes of SBP (40, 41). It seems that most of the studies have been performed in subjects with hypertension or in special situations favouring a metabolic alteration. This is the case of the studies carried out by Basu et al. or Grassi et al., where subjects were diabetic or were subjected for an atherosclerotic environment. In those studies, cocoa flavanols were able to reduce the SBP levels (38, 39). However, there are studies observing an increment of SBP after cocoa intake compared to the treated group. In this sense, De Gottardi et al. observed that blood pressure increased in cirrhotic patients after 30 min of consuming a liquid meal containing 0.55 g of dark chocolate/kg body weight (42). Similarly, West et al. assessed the acute effect of consuming 22 g of cocoa (814 mg flavanol) following the regular intake of cocoa during 4 weeks. These researchers observed a higher SBP at 2 h post-consumption in comparison with a flavanol free group, although no differences were found in blood pressure after the regular cocoa intake (43). The increment of SBP after cocoa intake could be attributed to theobromine content, which is the most important methylxanthine in cocoa, and although it does not have the same stimulating effect as caffeine, it has similar properties such as the stimulation of heart rate (44, 45).

More important is the result obtained concerning SBP when the differences between both postprandial tests (AUC_Postprandial 2 and AUC_Postprandial 1) were compared between both groups. Interestingly, a greater reduction of postprandial SBP was observed in cocoa consumers after 4 weeks of daily cocoa consumption independently of body weight loss and controlled for baseline SBP levels, suggesting that postprandial reduction of SBP could be affected by the daily consumption of cocoa extract. Although the subjects of the study were overweight/obese, overall the population of the study resulted normotensive. It is well known that obesity predisposes to suffer from hypertension (46). However, according to scientific evidence not all obese subjects are necessarily metabolically altered or hypertensive. Those subjects are known as ‘metabolically healthy obese’ (47, 48). This term refers to subjects suffering from obesity without metabolic alterations (48). Although the population of this study is normotensive, the energy-restricted diet was able to reduce SBP levels reporting a higher reduction in cocoa-supplemented group. This result supports the blood pressure–lowering properties of cocoa flavanol intake in obese individuals without current hypertension (37).

In our previously published data, subjects supplemented with 1.4 g/day of cocoa extract and the control group reduced significantly fasting SBP levels after the 4 week intervention. However, no differences were found beteween them (13). Thus, in that case, the reduction of fasting SBP levels was attributed to the weight loss diet followed by the volunteers during the 4 weeks and not to the cocoa consumption (13). However, in this research, the postprandial SBP was assessed, revealing that it may be affected after a regular consumption of the cocoa extract (7, 8).

On the other hand and as expected, a reduction was observed in the AUC of routine blood biochemical markers, but without differences between groups. This result should be attributed to the weight loss obtained after following the 15% energy-restricted diet (49).

Importantly, the measurement of food-derived metabolites in plasma is an useful method to assess the bioavailability of nutrients (23) and the compliance of food intake (10, 50, 51). In this study, 14 cocoa-derived metabolites were analysed in plasma. Ten metabolites were detected and quantified in cocoa consumers and 5 in the control group. As expected, cocoa metabolites in the plasma samples of cocoa consumers were detected during both postprandial tests, and the absence or significantly lower concentration of those metabolites were detected in control group. This outcome demonstrated the bioavailability of cocoa extract flavanols within the ready-to-eat meals as well as the compliance of the volunteers during the intervention. In accordance with other studies, most of the metabolites were phase II metabolites such as catechin and epicatechin-sulphates, methylates, and glucoronidates (52) but also alkaloid metabolites were detected in both groups. Epicatechin and catechin were not quantifiably intact after 1 h of the cocoa extract consumption, probably due to a rapid metabolisation in sulphates, methylates, and glucoronidates. Interestingly, the procyanidin B2 was detected in plasma of cocoa consumers (24).

Focussing on the cocoa group, 3-methylxanthine and theophylline levels were significantly in higher concentrations in the second postprandial period. This phenomenon could be due to the half-life of theobromine and derivatives in the circulation. While the half-life of flavanols in plasma varies between 6 and 8 h with a maximum concentration at 1–2 h post-consumption (24), the half-life of theobromine and derivatives varies between 7.5 and 10 h, with a maximum concentration at 2 h post-consumption (53). The presence of some cocoa-derived metabolites in the control group could be explained because of the presence of other flavanol sources in the diet such as apples, pome fruits, etc. The concentration of those flavanols in subjects from control group was in significantly lower amounts, in accordance with the results obtained in other studies (50). The metabolites reported in this study have been already identified in other cocoa-related studies (25, 50, 54).

Finally, it is important to highlight the strengths and limitations of the present investigation. First, a crossover design would have been appropriate because each crossover patient serves as his/her own control reducing the effect of confounding covariates. Nevertheless, the crossover design was declined because the volunteers would present a weight loss due to the prescribed energy-restricted diet. In this sense, weight change would have been a confounding factor since weight loss would not have been the same in the first phase comparing with the second phase of the crossover. For this reason, parallel study was designed. Second, blood pressure measurements were not performed in series of three consecutive measurements in order to avoid overwhelming the volunteers during the postprandial period, which can be considered as a weakness of this study. Third, the assessment of cocoa-derived metabolites in plasma is considered a reliable method to evaluate the nutritional compliance of the volunteers. Nevertheless, not to consider the effect of cocoa extract beyond the 180 min post-consumption is a limitation of this study.

## Conclusions

The intake of cocoa extract within an energy-restricted diet during 4 weeks revealed a higher reduction of postprandial AUC of SBP when compared to control group. In addition, cocoa-derived metabolites were detected in plasma of cocoa consumers suggesting the bioavailability of cocoa compounds within the ready-to-eat meals. Overall, this research provides a new evidence to understand the role of cocoa flavanols on postprandial cardiometabolic markers including blood pressure.

## Authors’ contribution

II-B contributed to the design of the study, was involved in the fieldwork, data collection, analysis, and writing of the manuscript. MS and AA-A contributed to the analyses of cocoa-derived metabolites and editing the manuscript. JAM and MAZ were responsible for the general coordination, design, interpretation of the data, financial management, and editing of the manuscript. All the authors actively participated in the manuscript preparation, as well as read and approved the final manuscript.

## Supplementary Material

Cocoa extract intake for 4 weeks reduces postprandial systolic blood pressure response of obese subjects, even after following an energy-restricted diet
