# Supplementary material for: Cocoa extract intake for 4 weeks reduces postprandial systolic blood pressure response of obese subjects, even after following an energy-restricted diet
Source: Food Nutr Res. 2016 Mar 31;60:10.3402/fnr.v60.30449. doi: 10.3402/fnr.v60.30449 (PMC4818356; doi:10.3402/fnr.v60.30449)
Supplement: Cocoa extract intake for 4 weeks reduces postprandial systolic blood pressure response of obese subjects, even after following an energy-restricted diet [file FNR-60-30449-s001.docx]

**SUPPLEMENTAL MATERIAL**

**Supplemental Table 1** Optimized selected reaction monitoring conditions for the analyses of cocoa derived metabolites by HPLC–MS/MS.

| **Compound** | **MW** | **Quantification** | | |  | **Confirmation** | | |
| --- | --- | --- | --- | --- | --- | --- | --- | --- |
|  |  | **SRM _1_** | **Fragmentor (V)** | **Collision energy (V)** |  | **SRM _2_** | **Fragmentor (V)** | **Collision energy (V)** |
| Catechin | 290 | 289 > 203 | 120 | 20 |  | 289 > 245 | 120 | 25 |
| Epicatechin | 290 | 289 > 245 | 130 | 10 |  | 289 > 203 | 130 | 20 |
| Procyanidin B2 | 578 | 577 > 425 | 130 | 10 |  | 577 > 407 | 130 | 30 |
| Methyl-catechin-glucuronide | 480 | 479 > 303 | 160 | 20 |  | 479 > 289 | 160 | 20 |
| Methyl-epicatechin-glucuronide | 480 | 479 > 303 | 160 | 20 |  | 479 > 289 | 160 | 20 |
| Epicatechin-glucuronide | 466 | 465 > 289 | 140 | 20 |  | 465 > 203 | 140 | 40 |
| Catechin-sulphate | 370 | 369 > 289 | 140 | 20 |  | 369 > 245 | 140 | 20 |
| Methyl epicatechin-*O*-sulphate | 384 | 383 > 303 | 140 | 20 |  | 383 > 245 | 140 | 10 |
| Epicatechin-sulphate | 370 | 369 > 289 | 140 | 20 |  | 369 > 245 | 140 | 20 |
| 3-*O*-methyl-epicatechin | 304 | 303 > 137 | 180 | 20 |  | 303 > 245 | 180 | 10 |
| 3,7-dimethyluric acid | 196 | 195 > 124 | 110 | 20 |  | 195 > 180 | 110 | 20 |
| 3-methylxanthine | 166 | 165 > 122 | 110 | 20 |  | 165 > 150 | 110 | 20 |
| 1-methylxanthine | 166 | 165 > 108 | 40 | 20 |  | 165 > 122 | 40 | 20 |
| Theophylline | 180 | 179 > 164 | 40 | 20 |  | 179 > 122 | 40 | 20 |

Abbreviations: MW: Molecular weight; SRM : Selected reaction monitoring

**Supplemental Table 2** Retention time, calibration curves, linearity, process efficiency, limits of detection and limits of quantification

| **Compound** | **RT**  **(min)** | **Calibration curve** |  | **Linearity**  **(nM)** | **R^2^**  **(%)** | **Process efficiency**  **(%)** | **LOD**  **(nM)** | **LOQ**  **(nM)** |
| --- | --- | --- | --- | --- | --- | --- | --- | --- |
| Catechin | 6.3 | y = 0.1493x |  | 11.1-3100.6 | 0.998 | 102 | 3.3 | 11.1 |
| Epicatechin | 6.8 | y = 0.1459x |  | 13.5-3100.6 | 0.999 | 80 | 4.0 | 13.5 |
| Procyanidin B2 | 6.6 | y = 0.2267x |  | 2.9-864.3 | 0.981 | 76 | 0.9 | 2.9 |
| 3,7-dimethyluric acid | 2.4 | y = 0.753x |  | 53.6-112170.2 | 0.981 | 71 | 16.0 | 53.6 |
| 3-methylxanthine | 2.8 | y = 0.932x |  | 211.9-99192.4 | 0.945 | 76 | 63.6 | 211.9 |

Specifications for the determination of phenolic compounds in spiked plasma samples.

Methyl-catechin-glucuronidate and catechin-sulphate were quantified using the calibration curve of catechin. Methyl epicatechin-glucuronidate, epicatechin-glucuronidate, methyl-epicatechin–*O*–sulphate, epicatechin-sulphate and 3-*O*-methil-epicatechin were quantified using the calibration curve of epicatechin. 1-methylxantine and theophylline quantified using the calibration curve of 3-methylxanthine.

Abbreviations: Retention time (RT, min), linearity, process efficiency (%R**^2^**), LOD: Limit of detection; LOQ: Limit of quantification.
